# The *Medical and Physical Journal* and the construction of medical journalism in Britain, 1733–1803

**DOI:** 10.1017/mdh.2024.19

**Published:** 2024-10

**Authors:** Alan Mackintosh

**Affiliations:** Centre for the History and Philosophy of Science, University of Leeds, Leeds, LS2 9JT, UK

**Keywords:** Medical journals, *Medical and Physical Journal*, *London Medical Journal*, Richard Phillips, Surgeons, Medical readers

## Abstract

Medical practitioners, inevitably scattered across the country, need frequent periodicals to communicate the latest medical information. Journals are an essential component of the infrastructure of modern medicine, yet they were slow to achieve firm roots in Britain during the eighteenth century, with few sustained quarterly periodicals and the only attempt at a monthly lasting a year. Then in 1799, Richard Phillips, owner of *the Monthly Magazine*, published the *Medical and Physical Journal*, the first sustained monthly medical journal, which lasted for thirty-four years. Ever since, Britain has never been without a monthly or weekly general medical journal. Responding to the need for a strong commercial focus, the *Journal* used a magazine format which blended reviews and abstracts of already published material with original contributions and medical news, and it quickly achieved a national circulation by close engagement with all types of practitioners across the country.

Contrary to the historiography, the *Journal* was distinctly different from the contemporaneous monthly science journals. The key to success was two-way communication with all practitioners, especially the numerous surgeons and surgeon-apothecaries who were increasingly better trained and confident of their status. Much of the content of the *Journal* was written by these readers, and with rapid, reliable distribution and quick publication of correspondence, controversial topics could be bounced back and forth between all practitioners, including the distinguished. Initially, the editors tried to maximise circulation by avoiding any controversy, but this started to change in the first few years of the next century.

Medical journals are an essential tool of modern medicine, providing a medium for formulating, circulating, and authorising medical knowledge. Opportunities to do this by oral communication are limited by the spread of medical professionals across the whole population. Books and single pamphlets can be effective print sources, but regular periodicals, on paper or more recently electronically, are the staple for communication within medicine, aiming to maximise the public’s health and, potentially, the practitioners’ income. In the early eighteenth century, some of the virtues of medical periodicals were already recognised, especially their ability to spread concise contributions quickly across a wide audience.

Many good and useful remarks and discoveries are lost, by the unwillingness of some ingenious men to appear in print, and by others having neither the time nor inclination to compose a sizable treatise, who would communicate necessary and beneficial observations to the World, if they had a proper opportunity to do it in a sheet or two.^1^

Yet the early development of medical periodicals in Britain was characterised by many attempts and not much success. How did the periodicals develop from erratic peripheral supplements to essential components of the medical infrastructure? Several London booksellers published an expanding and profitable list of medical books during the eighteenth century, and the increased numbers and improved education of medical practitioners in Britain would seem to provide fertile soil for medical periodicals. The basic problem was that the earliest journals limited their range of medical information and concentrated on the upper echelons of the profession. Rather than disseminating all relevant medical knowledge to any practitioner who might be interested, they focussed on either original observations or reviews and abstracts of previously published material, and they were commonly framed as the product of a learned society with erudite articles, often lengthy, for classically educated physicians. However, survival depended not on the quality of the writing but on sufficient sales to generate profits for the publisher, and this was only achieved once the journals adopted an unabashed commercial milieu, maximising circulation by reflecting the interests and needs of all branches of the profession.

This successful market re-orientation was the result of many changes, but three stand out. One was the incorporation of the increasingly better-educated, better-trained, and more confident surgeons as readers, contributors, and editorial staff: a sufficient circulation was difficult without the full involvement of the largest of the three branches. The second was the adoption of a magazine format, which combined original articles, reviews, medical news, and other material to provide a varied diet of concise information, much of it written by readers, thus fulfilling the comprehensive needs of these professional men. The vast majority of potential readers dealt with all aspects of medicine, and for their livelihood, they needed to know something about everything. The third was the provision of a ‘noticeboard’ to allow anybody to submit a comment or observation for publication, enabling, for the first time, full discussion by all practitioners across the country. Other contributing factors to be considered include regular monthly publication, an experienced publisher, named and interactive editors, improved national communications, and competition among practitioners.

Casting a wide net, LeFanu and Loudon listed thirty-four British medical periodicals published prior to 1799, but many were ephemeral and most of the others were erratically timed, occasional, transactions of learned societies, which cannot be regarded as true periodicals as defined in the Oxford English Dictionary.^2^ Only four regularly published medical periodicals had survived over three years, and the only attempt at a monthly had lasted a year. Then in 1799, the *Medical and Physical Journal* ushered in a new era of British medical periodicals by combining all the features required for a substantial circulation and so producing an issue every month for thirty-four years. Competitors followed, and since 1799, Britain has never lacked at least one weekly or monthly medical journal. However, despite this central role, the *Medical and Physical Journal* has rarely featured in the historiography of early medical journals during the last thirty years.

A brief summary of the early development of medical periodicals starts with the transactions of learned societies, such as the Royal Society, and the general commercial monthlies, such as the *Gentleman’s Magazine*, which printed articles on medical topics for the attention of both medical practitioners and the educated lay public.^3^ Starting with the annual *Medical Essays and Observations* in 1733, a few commercial, annual or quarterly, medical periodicals, and the sporadic transactions of medical organisations, kept some practitioners erratically informed. For most of their content, these publications usually chose either the format of original contributions or the format of reviews or extracts of existing works. From the 1790s, bimonthly or monthly commercial medical journals added in notices of events and two-way communication between readers and the editor, or between the readers themselves. Some of the journals in this period combined the two earlier formats into a magazine structure. From the 1820s, weekly journals such as *The Lancet* and the *Medical Gazette* could provide almost immediate reporting and comments for events relevant to the medical profession.

Several authors, starting with LeFanu in 1938, have documented the bibliographies of early English-language medical journals on both sides of the Atlantic.^4^ These impressive bibliographic publications, which record hundreds of medical journals (many only surviving for a handful of issues), provide little detail on the content of the journals, and even less on their aims, achievements, and interactions with the medical profession. Then in 1992, papers collected in a book on medical journalism, *Medical Journals and Medical Knowledge*, made a promising start on the detailed exploration of medical journals published before *The Lancet*, although most of the papers dealt with later periods.^5^ In the book, Roy Porter detected a ‘trend towards true journalism’ in the *Medical and Physical Journal* but avoided detailed analysis, and the Loudons recognised the importance of the magazine format to the same journal, without further exploration.^6^ A few studies over the subsequent thirty years have explored some specific aspects or have briefly discussed the early journals, but none have mentioned the *Medical and Physical Journal.* ^7^ In a recent collection of papers on nineteenth-century medical journalism, the editors, Sally Frampton and Jennifer Wallis, regretted the shortage of substantial work on early medical journals, which they partly attributed to a separation between medical historians and journal historians.^8^

Medical periodicals have also been incorporated into explorations of early science journals, but the common perception of the medical periodicals as a specialised version of the latter is misleading.^9^ The commercial science journals at the end of the eighteenth century and the *Medical and Physical Journal* were all octavo monthlies in a magazine format, but the medical journal was distinctly different in aims and content. At this time, medicine was a well-established profession, whereas science as a recognised profession did not yet exist. Clearly, the two categories of periodicals overlap as some of the topics would be of mutual concern, but the audiences were dissimilar, with medical practitioners seeking constructive information and debate on a variety of topics to promote their livelihood and the science journals recording new findings, often already published, for a wider group of ill-defined readers. In this period, the two-way relationship of the *Medical and Physical Journal* with its readers was not mirrored in the science journals.

At first glance, the shortage of historical studies of the early British medical periodicals is surprising, as they are central to both periodical history and the history of British medicine. In the former, medicine, the arts, and agriculture were the subjects for the first specialist commercial periodicals which explored the needs of a specific group and attempted to make money for the publisher.^10^ All periodicals are a production of their time. For the science journals, James Secord has observed that historians often take for granted the existence of specialist journals as useful sources, unaware of the journals’ capacity to alter the material.^11^ Similarly, in the history of medicine, reliable material is routinely extracted from accessible periodicals, apparently without awareness that the nature and aims of the early journals adjust the information provided and so transmute our understanding of British medicine.

Practical issues have hampered the study of the early medical periodicals. One problem is a shortage of suitable archives outside the periodicals themselves: most periodicals were not long-lasting, the owners could be consortia of booksellers without individual responsibility, and the editors could be genuinely anonymous. A notable exception is one of the earliest periodicals, *Medical and Philosophical Commentaries*, founded in 1773, and its successor *Annals of Medicine*, where the records of the publisher, the first John Murray, have provided much detailed information for a revealing book.^12^ With other journals, we are mostly reliant on the periodicals themselves, and for this study, they are the main actors, with the owners and editors providing a supporting role where known. This introduces a major problem: the size of each volume, the dense layout of the pages, the rambling literary style, and the old, now difficult-to-understand medical theories are a challenge to anybody who does not have several years to spare. Each octavo volume commonly contained around five hundred pages of packed type accompanied by minimal headings and only occasional illustrations. Detailed examination of every page of even the few sustained journals is not practical. In this investigation, selected years of the journals have been studied in detail, together with an inspection of volumes from other years. The emphasis is on the more successful journals which would have a larger readership and a greater influence on later editors and publishers. The selection includes the *second* calendar year of publication for each major journal: in the first few months, the articles may be atypical and reader contributions have not had time to develop, whereas in the second year, the intentions of the editors and the feedback from the paying customers combine to represent more clearly the longer-term style and content of the journal.

In this paper, I first explore the development of British medical periodicals before 1799. Content varied, but most periodicals chose a dominant format of either original contributions or reviews, summaries, and extracts of existing publications. Maintaining even annual publication proved difficult for much of the eighteenth century. Then *Medical and Philosophical Commentaries* in the 1770s and the *London Medical Journal* in the 1780s for a time became stable quarterlies, introducing some of the novelties required for commercial success. Two monthly medical journals started in March 1799, with one, the *Medical and Physical Journal*, establishing a successful magazine format by mixing both types of content with letters to the editor, brief items of news, and anything else that practitioners might want to read. I explore the reasons for its success and for the failure of the rival new monthly, the *London Medical Review and Magazine*, and particularly the rapid acceptance of the *Journal* by all types of practitioners and its provision of a noticeboard for debate across the country. I contrast medical journals with commercial science journals which also emerged in the 1790s. In the final section, I briefly introduce some of the developments of the *Medical and Physical Journal* after 1803.

## Publishing eighteenth-century medical periodicals

Two periodicals, both started in 1665, set the pattern for science and medical journals over the next century and a quarter.^13^ *Philosophical Transactions*, based on the work of the Royal Society, was a wide-ranging collection of original information from across natural philosophy and physic. The *Journal des Scavans* predominately printed reviews of published books and pamphlets, including many devoted to natural philosophy and physic. This pattern of either concentrating on original findings and opinions or providing access to existing material through reviews and extracts was not rigid, and some periodicals provided both. However, most scientific and medical journals up to the end of the eighteenth century concentrated on one of these broad categories. For medical periodicals in Britain, the result was presented in two formats: one filling the pages predominately with original communications from medical practitioners and natural philosophers and the other with summaries, reviews, and reprints of existing works from home and abroad, often accompanied by a few original communications.

The two formats required different editorial techniques. With original communications, editors had to assemble material and only write a modest amount for publication; however, concerns were often expressed about the limited supply of articles hampering regular publication. By contrast, utilising existing works assured a copious supply of material, but it necessitated substantial reading, and the writing of reviews and summaries, by the editors or their friends. Until the early nineteenth century, medical editing was very much a part-time activity for established practitioners, and editors commented on the difficulty of combining the necessary time with their other commitments. When the *Medical and Physical Journal* combined these two formats into a new magazine format, two editors, now called ‘conductors’ with a more substantial role, were required.

For most of the eighteenth century, British medical periodicals usually had a lead editor, but he remained anonymous, while a learned society apparently ran the journal without concern for commercial success. Similarly, mid-eighteenth-century literary journals were often nominally run by a ‘society of gentleman’, despite having a single editor.^14^ The presumed communal judgement of a medical society would bolster the authority of the contents and protect the editor from personal attacks.^15^ Often without a specific name or published office holders, most of these medical societies do not seem to have had a separate existence beyond assisting the editor. All the medical periodicals that achieved sustained *regular* publication in the second half of the eighteenth century were commercial investments by well-known bookselling publishers, and not, as they sometimes implied, solely for the dissemination of medical knowledge without any profits being considered.

The first sustained medical periodical in English, *Medical Essays and Observations*, started in Edinburgh in 1733 as an annual with an original communications format. It only ran to five volumes, but its aims, contents, and regular publication denote the birth of British medical periodicals. The opening volume was dedicated to the President and Fellows of the Royal Society, and the editors made it clear that the new periodical aimed to imitate *Philosophical Transactions* as a repository of durable knowledge, but specialising in medicine.^16^ In its first few pages, *Medical Essays* set out its aims and methods, including eleven detailed points on the content and style of submitted articles; these included an element of peer review with the editors explaining that a paper might be revised and the author questioned before acceptance, which was not guaranteed.^17^

*Medical Essays* arose from a substantive Edinburgh medical society, originally created in 1731 to publish histories from the patient register of the newly opened Edinburgh Royal Infirmary.^18^ Alexander Munro *primus*, surgeon and professor of anatomy, was the secretary and editor. The society quickly decided to publish a wider range of cases and other original articles. But the society then faded away, and from 1733, Munro had sole, but anonymous, editorial responsibility. The first volume of 330 pages was dominated by original articles (eight essays and twenty-four case reports), which were followed by some brief descriptions of other medical advances.

The journal managed three of its intended annual volumes. The fourth was delayed by a year as a result of Munro’s nearly fatal fever at the end of 1735, and the last volume appeared in two parts in 1742 and 1744.^19^ However, it did not stop making money for publishers. In common with other early medical journals, it was partly a periodical and partly a series of books. Long after fresh volumes had ceased, lightly revised editions of the existing volumes were issued by various publishers in Edinburgh and London up to a fifth edition in 1771. Thirteen years after the last original volume, the *Monthly Review* regretted its closure – ‘But as these volumes are among the most valuable of the annals of physic, so their discontinuation (…) was a signal loss to every branch of the faculty’.^20^

The original communications format also appeared in two London publications which were both aimed at physicians – though their irregular and occasional appearances mean that neither can be regarded as a true periodical. One was *Medical Observations and Inquiries* (1757–84), which consciously followed the aims and methods of *Medical Essays and Observations.* The society of physicians which ran this periodical had no specific name, but some of its members were identified in the text, the most prominent being two Licentiates of the College of Physicians John Fothergill and John Clephane, joined by another, William Hunter, in later volumes. Again, the periodical paid homage to *Philosophical Transactions*, and also to publications of the French Academy, but explained that they were insufficient for the needs of medical practitioners.^21^ Each volume consisted of a single issue devoted to original communications with no items that would become dated. The editors clearly stated that publication would only occur when enough suitable articles had been received and only six volumes appeared over twenty-seven years. Similarly, the second publication, *Medical Transactions*, produced by the London College of Physicians, has more claim to be a published series rather than an early medical periodical. Like *Medical Observations*, it only consisted of original communications with a stated preference for argued theses rather than simple case reports.^22^ A mere six volumes were printed between 1768 and 1820.

The first English-language medical journal to achieve regular publication over several years contained some original communications but devoted most of its pages to the format of reviews, extracts, and abstracts of existing publications. Edited anonymously by Andrew Duncan senior in Edinburgh and published by Charles Elliot in Edinburgh and John Murray in London from 1773, the quarterly *Medical and Philosophical Commentaries* provided ‘a concise view’ of medical books and other publications, ‘saving time in reading and expense of purchasing books’.^23^ With its plentiful supply of publishable material, *Commentaries* was the first British medical periodical to sustain quarterly publication, as promised in the opening issue.^24^

In common with the original communications format, the review format started as a specialised version of existing more general publications. *Commentaries* acknowledged its debt to two predecessors.^25^ One was the Leipzig-based *Commentarii de Rebus* (1752–98), the pioneer scientific review journal. Every quarter it reviewed, from across Europe, scientific and medical books, dissertations, and journals, publishing in Latin to facilitate international access. The other was the British monthly literary journals, which aimed to review most books published in Britain.^26^ The two dominant literary journals in the early 1770s were the anti-establishment *Critical Review* and the Tory *Monthly Review*, and many of their reviews dealt with medical publications. During 1772, the year before the publication of *Commentaries*, the *Critical Review* published 47 reviews of medical works and the *Monthly Review* published 102.

For Duncan, reviews in his journal had a different purpose than those in the literary monthlies. Many of the articles in the monthlies were brief and aimed at allowing the reader to choose which books would be of interest. The reviews in the *Commentaries* were intended to provide practitioners with the relevant information so that the books did not need to be read.^27^ This style of review comprised a summary and assessment of the main findings of a publication, perhaps with some direct quotations, but with little discussion on the significance of the conclusions or the opinions of the authors. The *Commentaries* did not intend to be critical in the manner of a modern review:

As it is not our intention to offer any opinion with regard to the general characters of books, we shall, on every occasion avoid, as much as possible, either applauding or condemning any author.^28^

Each issue of *Commentaries* was divided into four sections and devoted most of its space to reviews and brief extracts of existing publications, together with some original communications, as long as they were concise.^29^ Analysing the second year as explained earlier, 60% of the 434 main pages from 1774 were devoted to the first section, ‘Account of Books’, and 16% to the second, ‘Medical Observations’. A major advantage of this format was that books written in the two major languages of European medical communication, Latin and French, were reviewed and summarised in English. A majority of the thirty-nine publications reviewed in 1774 were written in a foreign language (twelve in Latin and eleven in French).

Duncan was paid fifteen pounds for each issue, with Murray taking the profit for the London edition, and over a thousand copies were sold across the United Kingdom in the early years.^30^ Duncan confessed that his difficulty in finding time for the substantial amount of writing had delayed the publication of an issue, and this would probably happen again.^31^ The journal became an annual, entitled just *Medical Commentaries*, in 1780, selling around five hundred copies of the London volume under a new publisher. In 1796, it evolved into the annual *Annals of Medicine*, and it eventually became a long-standing quarterly, the *Edinburgh Medical and Surgical Journal*, in 1805, all under the lead editorship of Andrew Duncan and later his son, also named Andrew.

The longest-lasting quarterly medical periodical in the eighteenth century was the *London Medical Journal* (1781–91), initially following the review format, and was published in its first year by John Murray as an ambitious medical monthly – the first in Britain. One novelty was a named editor who was available for direct communication. Up to this time, British medical periodicals had been set in the context of a learned society of physicians, or physicians and surgeons, as discussed above, but many had a single lead editor. Thus, all five volumes of *Medical Essays* claimed editorship by a medical or philosophical society, but the first four volumes were edited anonymously by Alexander Munro.^32^ *Medical Commentaries* was nominally produced by a society in Edinburgh, though Andrew Duncan was at least recognised openly as the secretary to the society.

At first, the *London Medical Journal* adopted this traditional smokescreen of editing by ‘a society of physicians’. But by the end of the first six months, the situation was made clear:

One of their members, who was the first and most active promoter of this business, undertook the office of editor, to arrange the materials, superintend the printing of the work, exc, while others engaged to assist in the different branches for which they deemed themselves best qualified.^33^

Although that editor was not named, he was quickly known to be the experienced medical writer and London physician Samuel Foart Simmons. Simmons was tailor-made for the post; he was not only a prolific medical writer but also the author of the *Medical Register* in 1779, 1780, and 1783, an unofficial record of medical practitioners and institutions throughout England.^34^ A medical journal was now being edited by a well-known, identified London physician without the thin veil of an anonymous society, and the ability to write directly to the named editor seems to have encouraged communications from practitioners. However, the original high ambitions of the *London Medical Journal* had to be reined back after a year: in the future, the journal would appear quarterly, and Simmons announced that monthly publication ‘has been found to require more time than he can conveniently set apart for it’.^35^

The *London Medical Journal* became more closely engaged with all types of practitioners, a key component of commercial success, as it changed from a review format to predominately original communications. In its second year, the template was almost identical to *Commentaries* – not surprising as it started with the same publisher. Four years later in 1786, after Murray and Simmons had parted, Joseph Johnson and Simmons were producing an entirely different periodical. Apart from a short Catalogue of Books at the end of each quarterly issue, the contents were now undivided, with no reviews or medical news. Most of the volume was original case histories and essays, and the remaining space was filled with abstracts of previously published case histories and essays. In other words, each quarterly issue now consisted of original communications, topped up with similar material recently published elsewhere. Readers applauded the switch, though Simmons was initially concerned that an erratic supply of original communications could hinder the essential regular publication^36^ However, the change to a journal consisting solely of original communications was completed the following year.

Original communications must be submitted by somebody, and the mechanism for this was the involvement of readers across the country, especially the numerous surgeons and apothecaries, who could initiate their own topics or respond to published articles. The greater engagement is readily apparent when we compare the origins of communications to the *London Medical Journal* in 1786 with its second year in 1782. During 1782, four of only eight original communications from Britain were from physicians: three from surgeons and one from a well-known apothecary. During 1786, 36 of the more numerous 40 original communications were written in Britain, and 22 (61%) of these were from surgeons and apothecaries living outside London. Non-physicians had always sent some original communications to both general and medical periodicals, but the medical periodicals had retained a semblance of physician dominance. Now, the surgeons, with a little support from the apothecaries, were writing much of each issue – a necessary change for maximum circulation. According to Simmons’s own incomplete *Medical Register* for 1783, the 363 provincial physicians in England were vastly outnumbered by 2 801 surgeons and apothecaries.^37^ The number of physicians in Britain was insufficient for a good circulation, and a frequent sustained medical publication needed to be purchased by a substantial number of surgeons and apothecaries. The *London Medical Journal* continued to eschew a magazine format, and forceful medical debate across the country would have to wait for a monthly journal, but discussion was visible in its revised format during 1786: for example, an article on amputations by the Leeds surgeon James Lucas provoked a response from an Essex surgeon, who explained that the apparent errors had ‘enticed me, from my present obscure situation, to appear, for the first time, as a writer’.^38^

The *London Medical Journal* announced in 1790 that it would metamorphose into *Medical Facts and Observations*, still published by Joseph Johnson and edited by Simmons. This new publication would continue the original communications format, but it would come out irregularly as a single-issue volume when sufficient material was available, with Simmons claiming that he no longer had the time for quarterly publication.^39^ After a good start with six volumes published in the first five years, publication became less frequent with the final volume in 1801.

In the late eighteenth century, specialist periodicals devoted to agriculture, science, the arts, and medicine were proliferating as publishers realised the potential readerships among occupational or interest groups across the country.^40^ Such periodicals potentially provided their owners with a regular income from a smaller initial investment than a book.^41^ Most books had to be prepared and distributed without the publisher having a clear idea of any profit from sales over several years. A monthly octavo issue, often five to seven printed sheets generating 80–112 pages, would be cheaper to prepare than a quarto book of several hundred pages, and success would provide a predictable monthly income, with failure not proving too costly.

The next sustained medical periodical was the bimonthly *Medical and Chirurgical Review* (1794–1808). The editors were again nominally anonymous, but Henry Clutterbuck was generally known as the sole editor, according to his later biographers.^42^ As the title implies, the *Review* was a compendium of medical literature with descriptions of new publications accompanied by extracts of predominately foreign books and journals. In its first few years, it did not publish letters or other original contributions. No ‘society’ was reported as being involved, and Clutterbuck was able to maintain the hard work of editing – apparently single-handedly – six issues a year until 1806, and even for a final year of monthly issues in 1807. Then in January 1808, the periodical closed for unspecified reasons, and he devoted his career to teaching and medical practice in London. He died a well-known physician in 1856, and the passage of half a century meant that his effusive obituaries mentioned frustratingly little about his time as an editor.

After Simmons’s successful expansion of original communications from all branches of medicine in the *London Medical Journal*, a dominant review format with anonymous authors seems contrary. However, as we shall see, the *Medical and Chirurgical Review* obtained a significant national circulation by the beginning of the next century. One reason for this success was that the *Review* had a louder voice than many of its predecessors or contemporaries. Specific ‘editorials’ are a nineteenth-century concept, but Clutterbuck introduced a mild version by publishing a ‘General Review of the State of Medicine’ in his first volume and, with adjustments in the title, in subsequent volumes.^43^ This article allowed Clutterbuck to cautiously expound his views on the state of medicine and the advances in related sciences, especially chemistry, before going on to summarise the contents of that volume.

The *Review* had two additional advantages. First, the editor was a surgeon who could understand the practical needs of the all-important non-physician readers. Clutterbuck was admitted as a Member of the London Corporation of Surgeons in 1790; though after ten years of editorship, he graduated MD from Glasgow and then styled himself as a physician.^44^ Second, from 1793, war with France hindered the movement of French and, later, other Continental publications and prevented visits to France. Medical literature did continue to cross the Channel, but it could be erratic and delayed, fuelling a market for summaries of foreign literature as provided by the *Review.* Thomas Boosey, the publisher of the *Review*, had been one of the prominent importers of French publications in general, and he may have recognised this demand.^45^ During the *Review*’s second year in 1795, fourteen of the fifty-nine substantial reviews and all ten of the more brief abstracts were derived from Continental publications. In 1800, this had risen to thirty-one out of one hundred reviews and sixty-one out of eighty-nine abstracts.

To summarise, until the end of the eighteenth century, medical periodicals had proved difficult to establish. A major reason for this was a separation between the expected provision of authoritative accounts for well-educated physicians and the practical needs of rank-and-file practitioners: either a modest supply of original articles from ‘societies of physicians’ or lengthy reviews of existing publications would only attract a limited audience. In the mid-1780s, the altered *London Medical Journal* offered a content more relevant to its potential readers, engaging the numerous surgeons and apothecaries. The *Medical and Chirurgical Review*, edited by a surgeon, achieved a national circulation in the 1790s but without adopting the magazine format which was becoming common among the proliferating specialist journals. Then in March 1799, *two* publishers entered the market for a monthly magazine-type, medical periodical, combining the two earlier formats and seeking to involve all practitioners, both as readers and contributors.

## Creating a national monthly medical journal

At the beginning of March 1799, the first issue of the *Medical and Physical Journal*, 112 octavo pages with two coloured plates at a price of two shillings, was published by Richard Phillips, the controversial and prolific publishing bookseller in St Paul’s Churchyard.^46^ It was to appear, without exception, at the beginning of every month for the next thirty-four years. This section and the next will explore the content of Phillips’s new magazine periodical and show how an experienced publisher was able to produce a sustained monthly medical journal, popular with all branches of the profession across the country.

Throughout his career in Leicester and London, Phillips was a provocative, divisive, and, at times, radical figure, who was imprisoned for selling the *Rights of Man*, became high sheriff of London with a subsequent knighthood, fell into bankruptcy in 1810, and was allegedly involved in a fire insurance fraud.^47^ In 1799, Phillips was in the middle of a very productive period of publishing, helped by the rapid success of his general journal, the *Monthly Magazine*, started in 1796 with the assistance of Joseph Johnson.^48^ Phillips had the resources and the experience to produce and distribute a national medical magazine every month. Using ‘journal’ in the title is significant. This word had been commonly used in the titles of Continental medical and other publications, particularly the *Journal des Scavans* as far back as 1665, but it had appeared first in a British medical publication with the *London Medical Journal*, which was also a monthly periodical in its first year. In eighteenth-century English, ‘journal’ was often used for some form of daily record for private or official use.^49^ Its use in the title implies a greater immediacy than early periodicals described as a review, essays, or commentaries.

However, this new medical journal was not alone, and the rival journal emphasised its magazine structure by including the word in the title. The monthly *London Medical Review and Magazine*, apparently edited in the traditional manner by an anonymous group of London physicians and surgeons, was first published on 31 March 1799 by a consortium of five London booksellers.^50^ The earlier announcement of the intent to publish this monthly medical journal aimed exclusively at a readership of medical practitioners seems to have altered Phillips’s plans. According to a prospectus published at the end of the previous year, Phillips intended to publish a new monthly periodical on 1 February 1799, called prophetically the *British Medical Journal*, to provide a compendium ‘of all such matters of fact as lie scattered, at present, through a vast number of expensive and voluminous publications, as well foreign as domestic, and which deserve to be more generally known to the Medical Practitioner’.^51^ The new journal would be intended chiefly for practitioners, but it would ‘prove acceptable and useful to every class of readers’. No copies of this *British Medical Journal* have survived, and it is unlikely that any were printed.^52^ Instead, Phillips published the *Medical and Physical Journal* a month after the promised date of his earlier proposal, incorporating many of its planned characteristics, but with a new ambition.^53^ The preface to the first volume restated the earlier aim of providing a complete record of medical progress, but now its primary object was that ‘it should become a centre of communication’ between British practitioners.^54^ Non-medical readers were not mentioned. The rare absence of an issue of Phillips’s *Monthly Magazine* for February 1799 suggests substantial additional activity at his premises in St Paul’s Churchyard.^55^

In creating the new medical journal, Phillips drew extensively on his *Monthly Magazine*, which had always contained a strong medical component in its magazine format. The first editor of the *Monthly Magazine* was John Aikin, a practising physician, with contributions from John Wolcot (‘Peter Pindar’), a former Cornish medical practitioner, and the *Monthly Magazine* contained articles on medical topics from its inception in 1796.^56^ The first volume included a monthly tabulated report on the diseases in London and a long commentary on the legal attempts by the licentiates of the College of Physicians to obtain admission to its fellowship.^57^ In common with many monthly journals of the period, the *Monthly Magazine* continued to publish articles on medical topics that might be of interest to both lay and medical readers. But Phillips and his editors also sought a specific medical audience for this general journal. For example, in the volume for the second half of 1798, the last complete volume before the launch of the *Medical and Physical Journal,* the August issue published full details of the forthcoming winter courses of medical lectures that would only be of interest to medical readers.^58^ In the same volume, monthly reports of the diseases in London continued, Thomas Beddoes reported the establishment of his Pneumatic Institution, Dr Willich (soon to be the first joint editor of the *Medical and Physical Journal*) wrote three pages on the iniquities of ‘quack medicines’, and the early reports of the success of Perkins’s metallic tractors were discussed.^59^ Medical readers debated medical controversies, such as the cause of the recent yellow fever outbreak in New York and the use of emetics in resuscitation, though a footnote made it clear that the editor had no desire ‘to make our miscellany the vehicle of a medical controversy’.^60^ The *Monthly Magazine* was not a prototype of the *Medical and Physical Journal*, but it did publish some of the necessary components, especially a magazine format, letters from medical readers, regular reports of diseases, and announcements of lecture courses.

Phillips’s new title expressed a greater emphasis on precise medical knowledge and a wider audience than the vaguer name of *British Medical Journal.* ‘Medical’ in this period could refer to the whole of medicine, but it could also be used to emphasise a practical, empirical slant in health care. The title linked this pragmatic emphasis with ‘physic’, internal medicine derived from precedent and argument by classically educated physicians. Joining these terms with ‘journal’ suggested the provision of rapid access to both the theory and practice of medicine for the benefit of all physicians, surgeons, and apothecaries. It stole a small march on its rival by appearing at the beginning of March while the *London Medical Review and Magazine* emerged at the end of the month. As we shall see, the latter’s firmer identification with medicine in London may have had medical virtues, but at the expense of commercial success.

The *Medical and Physical Journal* was an immediate success, with purchasers and readers all over the country, in the substantial war-time armed forces, and across Europe and North America. The assertions of publishers are not always accurate, but in the third issue in May 1799, Phillips trumpeted that sales of the first two issues had ‘already considerably exceeded that of any medical journal hitherto published in this country’.^61^ The following year, the *Journal* was being circulated ‘universally’ through the Army and Navy, and a German translation was published in Leipzig.^62^ In addition to these claims in the journal, we have other indications of rapid acceptance by many medical practitioners. One piece of evidence is the day books of John Ware, a Whitehaven bookseller and publisher of the weekly *Cumberland Pacquet* newspaper.^63^ The geography is significant: Whitehaven is one of the most distant towns from London in England and not on the road to anywhere, so it would be one of the last places to get the latest news. The first preserved day book of August 1799 records that the sixth (August) issue of the *Medical and Physical Journal* was purchased by four local surgeons, and over the next three and a half years, the names of the surgeons changed, but Ware sold at least four copies every month. Four of the surgeons on the six-man medical committee for the local dispensary had a regular order for the journal at some time during this period.^64^ For comparison, the five-year-old *Medical and Chirurgical Review* was ordered by three to four surgeons every two months in the same period.

Another piece of evidence for the popularity of the *Medical and Physical Journal* is the borrowing registers of the medical libraries in Liverpool and Leeds.^65^ At Liverpool during 1801, the register’s first calendar year, the twelve unbound issues of the *Medical and Physical Journal* were borrowed on a total of nineteen occasions by seven practitioners, including four surgeons. For comparison, the six issues of the *Medical and Chirurgical Review* were borrowed on eight occasions by four practitioners. During the first calendar year of the Leeds register (1803), the *Journal* was borrowed on fifty-three occasions by fourteen practitioners (thirteen surgeons), and the *Review* was again somewhat less popular with seventeen borrowings by eight practitioners. Of course, these records are not a full picture of journal readership, but the records give a good indication of their relative popularity, and the total readership would have been higher. During the periods when the Leeds register seems complete, the *Medical and Physical Journal* was the most borrowed periodical up to 1815; the varying, non-chronological format of the Liverpool register prevents a similar comparison. Overall, the archival evidence coincides with the claims of the publisher: in the first few years of the nineteenth century, the *Journal* was the most popular medical periodical across Britain. The text contained no overt advertisements, except for publisher’s announcements, but the wrapper of each issue, now lost, may have included them. Contributors do not seem to have been paid routinely, but remuneration for at least the more formal theses cannot be excluded.

How did Phillips’s ‘conductors’ fill up the hundred pages or so of closely packed type in each issue of his new monthly journal? The key aims were variety and conciseness. In accordance with the methods of this study, the two volumes of the second year have been analysed in detail, a time when it was competing with the monthly *London Medical Review* and the bimonthly *Medical and Chirurgical Review*, with the *Annals of Medicine* publishing original observations in an annual volume. The *Journal* printed reviews of recently published medical books, pamphlets, and journals. During 1800, 171 of the 1 160 pages (15%) were devoted to 100 reviews, with 58 of the publications derived from the United Kingdom, 9 from France, and 28 from the rest of Europe. Although this was a substantial portion of the journal, it was considerably less than its competitors: for example, the *Medical and Chirurgical Review* by coincidence also published 100 reviews in its six issues during 1800, but they were lengthier, occupying 86% of its 595 pages. Many of the reviews in the *Journal* were limited in ambition, merely summarising the work and/or reprinting paragraphs. Some went further by commenting on the writing style, the handling of the topic, and the clarity of the conclusions, but only a few placed the work in a wider context or engaged in an analysis of the conclusions. The editors claimed to be avoiding criticism and to be allowing readers to judge for themselves the merits of a reviewed publication: substantial criticism had to be anonymous, and ‘editors, who give their names to the Public, cannot enter the lists of criticism’.^66^ However, this aim was not always achieved, and a few reviews did divulge comments that could be described in a later period as an editorial opinion. The development of editorial comment in early medical journals requires further study.

Another method of keeping readers up-to-date was the printing of short summaries and extracts from other publications, especially those published abroad. During 1800, excluding brief mentions and items that may have been original, 148 such summaries or extracts were printed. The majority of these pieces, with their sources nearly all acknowledged, concentrated on issues immediately relevant to medical practice, pharmacy, or chemistry, but they could stray into items of more general interest to medical practitioners, such as a dissection of an Australian platypus, the physicians’ report on the death of George Washington, and the banning of coffee in Sweden.^67^ The pieces aimed to provide readers with the latest information, concentrating on developments in continental Europe. Of the 148 summaries and extracts, 55 (37%) originated from France and 81 (55%) from the rest of Europe, with only 6 from the United Kingdom, 5 from the USA, and 1 without an identified origin. At a time when warfare was extensive across Europe, the *Journal* made a clear commitment to the internationalism of medical practice, reinforced by the appointment of a corresponding editor, Dr Noehden of Gottingen.^68^

An important new technique was for Phillips to retain direct control of his periodical, appointing editors as necessary to ensure continuity and commercial responsiveness. The initial conductors were Thomas Bradley, physician to Westminster Hospital, and Dr Willich, a German medical writer and contributor to the *Monthly Magazine.* By 1800, Willich had been replaced by the obstetric physician Robert Batty, a good choice for a time when many practitioners were seeking to expand their obstetric practice. Bradley and Batty remained as the principal conductors for seven years, and then Phillips was able to replace them to maintain control of his journal. Previously, several medical periodicals had in practice been the responsibility of a single practitioner, whatever the precise arrangements and the financial commitment. Publishing these earlier journals had similarities to publishing a book, with the editor providing much of the initial impetus and retaining intellectual ownership.^69^ For example, when Andrew Duncan fell out with John Murray in 1779, *Commentaries* moved with its editor to another publisher. Duncan conveyed the title back to Murray in 1783, and then moved it away again in 1786 when falling sales prompted Murray to negotiate a new deal.^70^ When Murray found it impossible to work with Samuel Foart Simmons, the *London Medical Journal* was transferred to another publisher, with Simmons continuing as editor.^71^ Similarly, the three monthly science journals started in the 1790s were owned by their editors, not their bookselling publishers.^72^ In contrast, the *Medical and Physical Journal* was undoubtedly Phillips’s creation. He changed one of his editors in the first year, and when he fell out with both editors, Bradley and Shearman, in 1810, he retained the journal and appointed two fresh editors.^73^ Phillips’s degree of day-to-day control of his medical journal is uncertain. We do know that Phillips personally supervised his *Monthly Magazine*, looking at all the correspondence, soliciting contributions, deciding what to publish, and revising the proofs.^74^

## Giving practitioners a noticeboard

Most of the rest of the *Medical and Physical Journal* was devoted to its key feature, the provision of a noticeboard where both distinguished and rank-and-file practitioners could supply the latest medical information and then debate its consequences. Much of the journal in 1800 was a stream of correspondence, and a few original theses, from practitioners all over the country and abroad. Like Phillips’s *Monthly Magazine*, a large part of the journal was written by its readers, not by its editors or a few expert contributors. By contrast, the *Medical and Chirurgical Review* printed no contributions from its readers in 1800, except for one letter of complaint and brief comments on two other letters received. Printing case histories, new operative techniques, considered opinions, and original theses sent in by readers and others was not new; it had been the main component of the original communication format since 1733. But earlier articles often aimed to be lengthy and erudite, commonly emanating from the leaders of the profession. For example, *Medical Facts and Observations* for 1797, the last volume before the *Medical and Physical Journal* started in 1799, printed ten submitted articles spread over a hundred pages, and their authors included the leading physicians Thomas Beddoes in Bristol and Richard Pearson in Birmingham, together with William Simmons (the prominent Manchester surgeon) and William Wright (physician to the forces in the West Indies). The communications from readers in the *Journal* during 1800 were more numerous and shorter, with many coming from rank-and-file members of the profession across the country. The swiftness possible in a monthly publication and the numerous provincial correspondents produced a livelier, more all-inclusive and more argumentative periodical, and it helped to ensure a wide circulation and profits for Richard Phillips. A notice to correspondents in the first volume confirmed that ‘valuable original communications’ were an ‘object of the first importance in establishing this journal’.^75^

The correspondence could be a case report, a new surgical technique, a description of a potential medical advance, thoughts on a topical medical issue, comments on an earlier article, a response to a letter from another reader, or a polished treatise. Their emphasis was on the practical details of medical practice, and the editors acknowledged their ‘infinite pleasure to observe the readiness and eagerness of their numerous correspondents’.^76^ A correspondent commended the wide range of material as a particular virtue, applauding the journal as a valuable miscellany for the country practitioner.^77^ Hot topics in 1800 included vaccination, treatment of burns, management of the retained placenta, opium for croup, urethral strictures, and digitalis in pulmonary diseases. In all, 220 items sent to the editors were published during the year (Table 1).

**Table 1.** Published correspondence to the *Medical and Physical Journal* during 1800

Observations and comments to tde editor	108
Case histories	91
Submitted treatises	14
Surgical techniques	7
**Total**	**220**

The submissions from readers in 1800 were apparently not subjected to peer review – only a little editorial review – and not much selection. We do not have any precise information on the number of letters received and the number accepted for publication, but probably most of the communications were published. The evidence for this comes from a small section at the end of each issue headed ‘To Correspondents’. It usually listed up to eight authors of correspondence received, but not included in that issue; their contribution was always published in later issues, usually within two months. In the same section, a small number of correspondents were informed that their contribution was not going to be included on various grounds, such as a letter ‘being in answer to a paper published in the journal with the author’s name, cannot be admitted without signature’, ‘the question proposed by JW cannot be discussed or answered in any periodical work; but we believe that any respectable physician will give him a satisfactory answer’, or even ‘Mr Martineau’s communication has been mislaid, and we shall be obliged to him for a copy of it’.^78^ It seems that no correspondence was ignored and that most was published quickly, with the excluded few requiring a printed explanation from the editors.

The publication of most correspondence is a check to any rosy ideas that the journal unleashed a pent-up torrent of observations and comments from practitioners. In comparison to other medical periodicals, the communications were numerous, but in the context of the number of practitioners in the United Kingdom, the quantity submitted is less impressive, especially as several practitioners had multiple letters published. Thomas Trotter, a well-known physician on matters of naval health, submitted five letters from Plymouth during the year, and Robert Kinglake, a Somerset physician, wrote four letters and a more complete treatise. The editors were not faced with a deluge of correspondence, and it seems that they did not select communications to any significant extent. Only a small fraction of practitioners contributed, but the *Journal* was a true miscellany, available to any practitioner, great or small, for observations on any topic.

An example of a topic which bounced back and forth during 1800 was the best management for a retained placenta after delivery.^79^ The January issue published a case from Davies, a London surgeon, recommending watching and waiting. In March, Peck, a Northamptonshire surgeon, disagreed and recommended early removal. In April, Davies responded that Peck had not appreciated all the causes of a retained placenta. The May issue contained three items on the topic. A case history from Wagstaffe, a London surgeon, supported Davies’s delayed approach; another case history from a Lancashire physician agreed with Peck’s early action; and an erudite letter from Squire, a London physician, claimed that both Davies and Peck were wrong. In June, Peck fired back with two letters written a month apart, the first disagreeing with Davies’s letter from April and the second disagreeing with Squire’s arguments in May, and Davies made it clear that he resented Squire’s attempts to link him to Peck. In July, a Suffolk physician gave his views on all the correspondence on the topic, recommending a cautious approach, and in August, Dr Kinglake of Somerset, as we have seen a frequent correspondent, favoured early, but gentle, action. Following two further cases from a Bradford surgeon, in October, a Lancashire surgeon wrote a nine-page letter attempting to give a definitive opinion. In November, a Wakefield surgeon disagreed with Kinglake, especially his techniques; this was rejected by Kinglake in a post-script to a letter on a different subject in the December issue. In all, the journal published fourteen letters and case histories from all over the country on the topic during 1800. The correspondence reveals a wide engagement in the best management of a retained placenta, and geographical separation was no longer a bar to forceful debate.

The importance of readers’ correspondence to a monthly medical journal is underlined by the experience of the *London Medical Review.* The two rival journals had looked rather similar at first, with the type in the *Review* less closely packed and better arranged. But the *Review* devoted most of its pages to reviews and attempted a more literary and authoritative style, with the editors providing additional discussion at the end of some reviews. The remainder of each issue of the *Review* was headed ‘Medical Correspondence’, with 68 reader communications during 1800 in a more polished style than the *Journal*’s 220 communications. Perhaps a reasonable number to start with, but only 20 came from outside London, and the editors of the *Review* inserted repeated appeals for more.^80^ The journal was edited by anonymous physicians and surgeons, with varying addresses printed for correspondence, and published by a changing consortium of up to ten booksellers: the overall impression is a lack of engagement between publishers, editors, and readers. John Ware, the Whitehaven bookseller, does not seem to have had any regular orders.

As a result, the *London Medical Review* did not live up to its founding intention of providing rapid and lively communications between all practitioners. By April 1801, it was in trouble, and its future would be reviews only, with all submissions from readers being redirected to the *Medical and Physical Journal.* ^81^ In July 1802, the *Review* threw in the towel. No more issues would be published ‘for want of sufficiently extensive encouragement’, and this was due to ‘the ascendency which a rival and contemporary publication, the *Medical and Physical Journal*, has deservedly acquired’.^82^

## Medicine for all practitioners, quickly and inclusively

For commercial success and therefore survival, the *Medical and Physical Journal* had to engage with the few thousand medical practitioners in Britain, and a significant proportion of these practitioners had to buy the journal each month, directly or through a subscription by a library, the armed services, or other organisations. Medicine was an old profession with an established position in society, and the journals at the start of the nineteenth century did not speak to particular groups and showed little interest in the status, organisation, or regulation of medicine. This section explores the professional and geographical range of the readers and correspondents, and it discusses further the reasons for their engagement with the journal. Some recent historiography has grouped early medical periodicals with contemporaneous science periodicals, but this is an oversimplification because medical needs differed from those of a science audience, and the medical publishing environment lacked the equivalent of the prestigious and authoritative *Philosophical Transactions.*

Engagement with practitioners across the country was helped by speed of distribution and the rapid publication of communications from readers. The first day of the month was the intended publication day for each issue. Whether this was always achieved is uncertain, but there is no evidence of significant delays in 1800 or later years. With the improved national communications, the *Journal* was usually available in the major centres within the first week of the month. Thus, the register for 1803 at the Leeds Medical Library, two days in a coach from London, reveals that a new issue was sometimes borrowed on the fourth day of the month, and usually within a week.^83^ The Liverpool register shows a similar speed of distribution. Distribution to the periphery of England would take longer. During 1800, timings for delivery revealed in the day book of John Ware in Whitehaven show a delay of three to four weeks to get the journal to its readers.^84^ Isolated Whitehaven was one of the farthest towns in England from London, and we can regard this timing as a rough upper limit.

The supply of original communications was reinforced by their rapid publication, far ahead of anything that might be sent to an annual or quarterly, if such a periodical would accept it. Most of the observations and comments to the editors (Table 1) were dated, and we can use this to document the short time from writing to publication. If we look at the 91 dated letters of observation and comment during 1800, coming from all over the United Kingdom and even from naval surgeons at sea, 48 (53%) were published within thirty days or less of writing, and 77 (85%) were published within fifty days or less. In the more populous parts of England, it was possible to read the latest issue, write a letter, and then get it published in the next month’s issue.

Contributions arrived from the whole range of practitioners. Some well-known practitioners, including leading physicians, contributed during 1800. For example, fourteen correspondents were later entered in the *Oxford Dictionary of National Biography*, including Edward Jenner, Gilbert Blane, Thomas Trotter, Thomas Beddoes, George Pearson, and even Henry Clutterbuck, the editor of the rival *Medical and Chirurgical Review.* Many of the distinguished correspondents also wrote books, but the *Journal* was an opportunity for more compact contributions. *Philosophical Transactions*, published twice yearly at this time, contained some medical papers, but in the absence of a regularly produced, authoritative periodical from a medical corporation, the *Journal* was able to record the views of the distinguished and print their replies to criticism.

However, the majority of correspondents were surgeons and apothecaries who were unknown outside their area (Table 2). One hundred and ninety-eight of the 220 correspondents provided an address and indication of their medical branch, and two-thirds lived outside London. Any style of letter or case report was acceptable. A Gloucester practitioner thought it important to send in a brief fourteen-line letter, which just recorded that he had initially been against vaccination but was now in favour, while the next letter was from a Sevenoaks surgeon describing in detail over three pages the techniques, appearances, and results of the vaccinations he had performed.^85^ The analysis of correspondents to the *Journal* not only indicates which practitioners were sufficiently engaged to submit material, but it also provides a rough guide to overall readership. The *Journal* was being read by many surgeons across the country. As we have seen, it was being purchased by Whitehaven surgeons in the early months of the periodical in 1799, and it was borrowed, a little later, by surgeons in Liverpool and Leeds. British practitioners could read about something relevant to the practice of medicine twelve times a year and then respond if they wished.

**Table 2.** Identified British contributors to the *Medical and Physical Journal* during 1800

	Number	Percentage of total
Provincial surgeons and apothecaries	79	40
Provincial physicians	57	29
London surgeons and apothecaries	42	21
London physicians	20	10
**Total**	**198**	

Surgeons had been increasingly engaged with the *London Medical Journal* during the 1780s, but their involvement with the *Medical and Physical Journal* was more than just numbers: surgeons and apothecaries were increasingly better educated, better trained, and more confident, with their successors becoming registered surgeon-apothecaries after 1815 or general practitioners as they came to be described. Would-be regular surgeons could still complete their five years of apprenticeship without any prior classical education and then practise without any additional training, but increasingly, non-physicians were classically educated and had attended London medical courses or Scottish universities for at least a year.^86^ Apart from a few specialist operative surgeons in medical centres, surgeons undertook the whole range of medical practice, and they wanted to be proficient across medicine, surgery, and midwifery. The conciseness and range of a magazine format would help them to be so, and many surgeons now had the education and experience not just to read a journal but also to participate in the subsequent discussion.

Also, surgeons were more confident of their importance within medicine.^87^ A few of the *Journal*’s surgical correspondents reflected an earlier attitude by first sending a contribution to a local physician for submission to the *Journal* ‘if you think it deserves a place’,^88^ but many were keen to submit their own assertive arguments, as we have seen with the multiple views on the management of a retained placenta. Some of the contributions may reflect a degree of self-interest rather than simply a desire to disseminate knowledge, as practitioners felt themselves to be in strong competition, and self-promotion of their experience in a periodical would do no harm. Phillips was fortunate in his timing because Edward Jenner formally published his new technique of vaccination nine months before the first issue of the *Journal.* ^89^ This technique provided a plentiful supply of content for the early volumes, with forty-eight contributions on vaccine-related topics, mostly original, printed during 1800. And a self-congratulation in an ‘advertisement’ during the same year boasted that ‘very valuable additions have been made to our knowledge of the Cow-pox; and that its discussion in our Journal has extended the benefits of this discovery to the world’.^90^ Vaccination was regarded across Europe as a revolutionary British technique, introduced by a surgeon using surgical skills, and so contributing to the growing confidence of British surgeons.^91^

How did the readership and the content of the *Medical and Physical Journal* compare with the expanding number of science journals? Much of the recent historiography of science journals has suggested that early medical and science journals shared many features.^92^ Science was increasingly regarded as the basis for medical advances, and no clear boundaries existed between the two genres. Eight journals devoted to a combination of science, the arts, and manufacturing were started in the 1790s.^93^ In common with the *Medical and Physical Journal*, the pioneering commercial science journals favoured a magazine format to maximise readership, named their editors, and sought original contributions.^94^

Yet, the *Medical and Physical Journal* exhibits important differences from the commercial science journals in its aims, content, and publishing status. It was supplying different information in a more intimate style to a specific type of reader. The nature of medical practice in this era meant that most practitioners could be confronted by an unexpected medical event at any time; so, for many, breadth of practical knowledge was more important than depth, and the main source of this knowledge was the experience of other practitioners. As we have seen, Richard Phillips and his editors responded to this need by including a wide variety of concise topics, often selected by their readers, and by promoting a relationship between the readers and editors. Most readers would find many topics of practical relevance in each issue. The readers of early science journals are hard to define, so delineating their requirements is difficult, and much of the historiography has focussed on the authorisation of knowledge and the building of scientific communities.^95^ The three available monthly science journals at the start of the nineteenth century addressed fewer topics in more depth – many of them already published elsewhere – with little attempt at reader involvement. Their readers would have only found an occasional article in each issue that would have directly affected their livelihood.

An examination of the issues for January 1800 demonstrates this difference. Wyatt’s *Repertory of Arts and Manufactures* contained eleven articles, five dealing with specific patents, and no original contributions; Nicholson’s *Journal of Natural Philosophy, Chemistry, and the Arts* had twelve articles of which six were original; and Tilloch’s *Philosophical Magazine* published fourteen articles, of which four were original. None of the science journals published any letters from readers, though original articles may have been submitted by post, and apart from brief comments on the sources of extracted articles, no words from the editors were printed. In contrast, the *Medical and Physical Journal* aimed at a closer relationship with its readers, printing forty-two articles in January 1800, mostly original and including useful information such as medical lecture times and the current diseases in London. Many of the articles were printed as letters to the editor, often addressing the editors at the beginning. The editors made brief comments, some quoted above, and provided a separate notice for correspondents in each issue. Overall, the science journals at this time aimed to be journals of record, whereas the *Medical and Physical Journal* went beyond this, seeking a two-way engagement with all ranks of practitioners.

The *Medical and Physical Journal* could attempt to communicate with all practitioners because the medical publishing environment lacked an equivalent to *Philosophical Transactions*, and so was less fractured. In science, the internationally prestigious quarto *Philosophical Transactions* only published contributions from fellows of the Royal Society or from other natural philosophers recommended by a fellow, and it maintained a distinction from the octavo commercial science journals, which contained articles by anybody, from the well-regarded in science to purely practical men.^96^ Topham’s description of the editors of commercial science periodicals as marginal men may be harsh, but two of them, William Nicholson and Alexander Tilloch, were unable to become fellows of the Royal Society.^97^ The editors of the medical journals were often among the leaders of the profession: six of the seven editors of the sustained regular periodicals discussed in this paper were in the top ranks, and only Henry Clutterbuck, the editor of the *Medical and Chirurgical Review*, could be described as a marginal figure at the start of his journal.^98^ As we have seen, well-known practitioners, both physicians and surgeons, submitted contributions to the *Medical and Physical Journal* and joined subsequent debates: the use of the noticeboard by *all* practitioners was important for the journal’s success. A vignette of the unified publishing environment of medical journals is Sir Joseph Banks, aristocratic president of the Royal Society and fierce defender of the exclusive status of *Philosophical Transactions*, personally submitting to the *Medical and Physical Journal*, with a covering letter, an original contribution from an unknown Lincolnshire physician.^99^

## Conclusion: dominance and progression

By 1803, the *Medical and Physical Journal* was the dominant British medical periodical, and indeed it was often simply recorded in the Register of the Leeds Medical Library as the ‘London Journal’. Richard Phillips had shown that ownership of a well-run monthly medical journal could be profitable – a solid foundation for later medical periodicals. Its magazine format engaged all classes of practitioners all over the country, and it appealed to the numerous and increasingly confident surgeons as well as physicians, helped by direction from named active editors, and the potential for participation in medical debate. Its monthly rival, the *London Medical Review*, had closed, the bimonthly *Medical and Chirurgical Review* concentrated on previously published material, and the annual *Annals of Medicine* from Edinburgh was not a direct opponent. The *Journal* had no monthly competitor apart from the brief reorganisation of the *Medical and Chirurgical Review* as a monthly in its final year until the editors, Bradley and Shearman, broke away to create the *New Medical and Physical Journal* in 1810. This new upstart – its name a tribute to its parent – lasted five years.

Did the provision of a persistent monthly periodical change British medicine? This a big question for a disparate grouping with varied agendas. Obviously, it speeded up and expanded the transmission of information and any subsequent discussion, as illustrated by the comprehensive debate on vaccination among enthusiasts such as Jenner and George Pearson, some forceful opponents, and practitioners from all over the country. Beyond the exchange of medical practicalities and theories, a regular journal could potentially advance a virtual community, or communities, among practitioners across the country, analogous to the virtual communities created by science journals.^100^ But this is not a straightforward topic, and it requires further exploration. The monthly issues allowed practitioners at a distance to learn the latest information, congratulate each other, exchange views, and achieve change. For example, efforts for medical reform from 1805 were accelerated by the rapid circulation of both the immoralities of current arrangements and the potential solutions. However, insulting another practitioner, whom you had never met and never would, was easy in print, and wounding comments on the character, morals, and lack of training of fellow practitioners enlivened the pages of many issues. The *Journal* undoubtedly brought some readers together, but it also pushed others apart.

Did the more informed medical profession change the *Journal*? This is a relatively simpler question with a distinct direction of travel, if not yet complete answers. Although Richard Phillips held radical views, the *Journal* in its first few years contained little or no evidence of them, with no mention of medical reform and minimal interest in the medical environment. Phillips was creating the market, and he, in common with Nicholson in his early science journal, presumably did not want to offend potential purchasers.^101^ Over the next two decades, journals became established medical vehicles, and they could appeal to particular medical communities, perhaps espousing medical radicalism.^102^ Several early readers understood the benefits of a national audience, and the *Journal* adapted to their needs by enlarging from just the art of medicine into the trade of medicine. For example, in 1802–03, space was allocated to an approved campaign: a group of London apothecaries and druggists, led by the surgeon William Chamberlaine, sought reforms to the 1802 Medicines Act, which had widened the taxation of medicines, resulting in vendors being persecuted by paid informers. The injustice of the Act, the initial meetings of the group, its proposals, and the subsequent meetings at The Treasury were described by Chamberlaine in thirty-seven pages over four issues, with the Act being superseded by the 1803 Medicines Act.^103^

The advent of national campaigning for medical reform that started in 1805 had a substantial influence on the *Journal* and later medical periodicals. Previous attempts at medical reform had concentrated on specific injustices, but the campaign led by Edward Harrison, with the active patronage of Sir Joseph Banks, proposed a national reform with prescribed training and registration for all practitioners.^104^ As the secretary of the organising committee, Henry Clutterbuck was a strong supporter of the proposed reforms, and he provided a great deal of space in his *Medical and Chirurgical Review* for Harrison to publicise information and favourable comments. Abandoning its earlier editorial attitude, the *Medical and Physical Journal* supported the principle of reform and published correspondence from both supporters and opponents.^105^ By 1807, both periodicals considered medical reform to be inevitable, and the *Journal* was drawn into an engagement with medical organisation. The *Journal*’s ‘Progress of Medicine in the Year 1806’, written in 1807 by William Royston, a London surgeon and subsequent editor, lamented the low status and remuneration of the profession and commended the efforts of Harrison.^106^ When medical reform was relaunched in 1812, the *Journal* was closely involved, providing space for the Association of Surgeon-Apothecaries to publicise its meetings, to report progress, and to acknowledge subscriptions.

Although the stance of the *Journal* changed in the years after 1803, Phillips and his editors were following the same principles that had ensured its initial success. By continuing to respond to the needs and requests of all practitioners, it maintained a good circulation of practical knowledge, which was simultaneously beneficial to the art of medicine, the publisher’s profits, and practitioners’ earnings. In particular, the better-educated and better-trained surgeons wanted, and indeed needed, to be well informed across the range of medical care and to participate in improving it. Increasingly, they also wanted to raise their status and income by reforming the provision of care, including the licensing of practitioners. For many years, the *Medical and Physical Journal* would aim to supply the needs of all practitioners and to maintain its position as a major monthly medical journal.

## Footnotes

1 *Medical Essays and Observations*, 1 (1733), v.

2

W.F. LeFanu , British Medical Periodicals. A Chronological List, 1640–1899, ed. J. Loudon , revised edn (Oxford: Wellcome Unit for the History of Medicine, 1984); ‘periodical, adj. and n, A magazine or journal issued at regular or stated intervals (usually weekly, monthly, or quarterly)’, Oxford English Dictionary Online, accessed 8 June 2022.

3

Roy Porter , ‘Lay Medical Knowledge in the Eighteenth Century: The Evidence of the *Gentleman’s Magazine*
’, Medical History, 29 (1985), 138–683884933 10.1017/s0025727300043970PMC1139505; Thomas Broman , ‘Periodical literature’, in M. Frascia-Spada and N. Jardine (eds), Books and the Sciences in History (Cambridge: Cambridge University Press, 2000), 225–38: 231.

4 LeFanu, *op. cit.* (note 2); W.F. Bynum and Janice C. Wilson , ‘Periodical knowledge: medical journals and their editors in nineteenth-century Britain’, in W.F. Bynum , S. Lock and R. Porter (eds), Medical Journals and Medical Knowledge: Historical Essays (London: Routledge, 1992), 29–48 ; Leslie Morton , ‘The growth of medical periodical literature’, in A. Besson (ed.), Thornton’s Medical Books, Libraries & Collectors: A Study of Bibliography and the Book Trade in Relation to the Medical Sciences, 3rd edn (Aldershot, Hampshire: Gower, 1990), 221–38; Myrl Ebert , ‘The Rise and Development of the American Medical Periodical 1797–1850’, Bulletin of the Medical Library Association, 40 (1952), 243–7614944975 PMC234302; James Cassedy , ‘The Flourishing and Character of Early American Medical Journalism’, Journal of the History of Medicine & Allied Sciences, 38 (1983), 135–506343476 10.1093/jhmas/38.2.135.

5
*Medical Journals and Medical Knowledge*, *op. cit.* (note 4).

6 Roy Porter, ‘The rise of medical journalism in Britain to 1800’, in *Medical Journals and Medical Knowledge*, *op. cit.* (note 4), 6–28: 17; Jean Loudon and Irvine Loudon, ‘Medicine, politics and the medical periodical, 1800–50’, in *Medical Journals and Medical Knowledge*, *op. cit.* (note 4), 49–69: 57.

7

David Kronick , ‘Medical Publishing Societies in Eighteenth-Century Britain’, Bulletin of the Medical Library Association, 82 (1994), 277–827920337 PMC225925; Iain Chalmers , Ulrich Trohler and John Chalmers , ‘ *Medical and Philosophical Commentaries* and its successors’, in J. Chalmers (ed.), Andrew Duncan Senior: Physician to the Enlightenment (Edinburgh: National Museums of Scotland, 2010), 36–55 ; Brittany Pladek , ‘“A Variety of Tastes”: *The Lancet* in the Early-Nineteenth-Century Periodical Press’, Bulletin of the History of Medicine, 85 (2011), 560–8622506433 10.1353/bhm.2011.0085; Michael Brown , ‘Bats, Rats and Barristers: *The Lancet* Libel and the Radical Stylists of Early Nineteenth-Century English Medicine’, Social History, 39 (2014), 182–209 25937694 10.1080/03071022.2014.905277PMC4373151; Sally Frampton , ‘The medical press and its public’, in D. Finkelstein (ed.), The Edinburgh History of the British and Irish Press, *Volume 2* (Edinburgh: Edinburgh University Press, 2020), 438–56.

8

Sally Frampton and Jennifer Wallis , ‘Reading Medicine and Health in Periodicals’, Media History, 25 (2019), 1–5 .

9

Jonathan Topham , ‘Anthologizing the book of nature: the circulation of knowledge and the origins of the scientific journal in late Georgian Britain’, in B.V. Lightman , G. McOuat and L. Stewart (eds), The Circulation of Knowledge between Britain, India, and China: The Early-Modern World to the Twentieth Century (Leiden: Brill, 2013), 119–52; Gowan Dawson and Jonathan Topham , ‘Constructing scientific communities’, in G. Dawson et al. (eds.), Science Periodicals in Nineteenth-Century Britain: Constructing Scientific Communities (Chicago: University of Chicago Press, 2020), 1–34: 21 ; Gowan Dawson and Jonathan Topham, ‘Scientific, medical and technical periodicals in nineteenth-century Britain: new formats for new readers’, in *Science Periodicals in Nineteenth-Century Britain*, *op. cit.*, 35–64.

10 Topham, *op. cit.* (note 9), 126.

11

James Secord , ‘Science, technology and mathematics’, in The Cambridge History of the Book, Volume 6, 1830–1914, ed. D. McKitterick (Cambridge: Cambridge University Press, 2009), 443–74: 444.

12 Chalmers, *op. cit.* (note 7).

13 Broman, *op. cit.* (note 3), 228–9; David Kronick , A History of Scientific and Technical Periodicals: The Origins and Development of the Scientific and Technical Press 1665–1790, 2nd edn (Metuchen, New Jersey: Scarecrow Press, 1976), 63, 280; Adrian Johns , ‘Miscellaneous Methods; Authors, Societies and Journals in Early Modern Britain’, British Journal for the History of Science, 33 (2000), 159–86.

14

Walter Graham , English Literary Periodicals (New York: Thomas Nelson, 1930), 204 .

15 Kronick, *op. cit.* (note 7).

16
*Medical Essays and Observations*, 1 (1733), iv; Aileen Fyfe et al., A History of Scientific Journals: Publishing at the Royal Society, 1665–2015 (London: UCL Press, 2022), 64 .

17
*Medical Essays and Observations*, 1 (1733), xvi. A form of peer review was used by *Philosophical Transactions* (Noah Moxham and Aileen Fyfe, ‘The Royal Society and the Prehistory of Peer Review, 1665–1965’, *The Historical Journal*, 61 (2018), 863–89).

18

Roger Emerson , ‘The Philosophical Society of Edinburgh, 1737–1747’, British Journal for the History of Science, 12 (1979), 154–91: 157.

19 H.D. Erlam, *Alexander Munro, Primus* (Edinburgh: Oliver & Boyd, 1954), 88; *Medical Essays and Observations*, 5 (1742), iii; Emerson, *op. cit.* (note 18), 168–70.

20
*Monthly Review*, 16 (1757), 542.

21
*Medical Observations and Inquiries*, 1 (1758), vii.

22
*Medical Transactions*, 1 (1768), vii.

23
*Medical and Philosophical Commentaries*, 1 (1773), 7.

24

William Zachs , The First John Murray and the Late Eighteenth-Century London Book Trade (Oxford: Oxford University Press, 1998), 179–80; Chalmers*, op. cit.* (note 7).

25
*Medical and Philosophical Commentaries*, 1 (1773), 6–8.

26 Graham, *op. cit.* (note 14), 204–14.

27
*Medical and Philosophical Commentaries*, 1 (1773), 6–7.

28
*Ibid.*, 9.

29
*Ibid.*, 10.

30 Chalmers, *op. cit.* (note 7), 37.

31
*Medical Commentaries*, 8 (1781), iv.

32 Emerson, *op. cit.* (note 18), 158.

33
*London Medical Journal*, 1 (1781), iii.

34 Helen Brock, ‘Simmons, Samuel Foart (1750–1813)’, *DNB Online*, accessed 5 October 2020.

35
*London Medical Journal*, 2 (1781), iv.

36
*London Medical Journal*, 7 (1786), iii.

37

Joan Lane , ‘The Medical Practitioners of Provincial England in 1783’, Medical History, 28 (1984), 353–71: 3556390030 10.1017/s0025727300036255PMC1140010.

38
*London Medical Journal*, 7 (1786), 225, 377.

39
*Ibid.*, 11 (1790), 400.

40 Dawson, *Scientific Periodicals*, *op. cit.* (note 9), 40.

41 Johns, *op. cit.* (note 13), 162–3; Broman, *op. cit.* (note 3), 234.

42

Anonymous , Authentic Memoirs, Biographical, Critical and Literary of the Most Eminent Physicians and Surgeons in Great Britain, 3rd edn (London: Sherwood, Neely and Jones, 1822), 318 ; Thomas Joseph Pettigrew , ‘Henry Clutterbuck’, in T.J. Pettigrew (ed.), Medical Portrait Gallery: Biographical Memoirs of the Most Celebrated Physicians and Surgeons (London: Fisher, 1839), 6.

43
*Medical and Chirurgical Review*, 1 (1794–95), 577–85. For most of its existence, the volumes of the *Review* started in July and ran to the following May.

44 Pettigrew, *op. cit.* (note 42), 3.

45

Jonathan Topham , ‘Science, print, and crossing borders: importing French science books into Britain’, in D.N. Livingstone and C.W.J. Withers (eds), Geographies of Nineteenth-Century Science (Chicago: University of Chicago Press, 2011), 311–44: 313.

46 The price increased to 2s 6d in September 1801. *London* was added to its title in 1815.

47 Pamela Clemit and Jenny McAuley, ‘Phillips, Sir Richard (1767–1840)’, *DNB Online*, accessed 23 November 2021; ‘Sir Richard Phillips, Knt.’, *Gentleman’s Magazine*, 14 (1840), 212–14; *Memoirs of the Private and Public Life of Sir Richard Phillips* (London, 1808); Thomas Jenkins, *Liberty of the Press* (London, 1808).

48

Helen Braithwaite , Romanticism, Publishing and Dissent: Joseph Johnson and the Cause of Liberty (Basingstoke: Palgrave Macmillan, 2003), 159 .

49 ‘journal, adj. and n.’, Oxford English Dictionary Online, accessed 23 November 2021.

50 Society of Physicians and Surgeons, *Prospectus of a New Monthly Publication* (London, 1799).

51
*British Medical Journal* (London, 1798), 2.

52 ‘An Eighteenth Century “British Medical Journal”’, *British Medical Journal*, 1 (1918), 183.

53 LeFanu’s listing of the *Medical and Physical Journal* as a continuation of *Medical Facts and Observations* is incorrect: it had different editors and publishers, and an appearance before the latter’s final demise.

54
*Medical and Physical Journal*, 1 (1799), i.

55 The sequence of issue numbers of the *Monthly Magazine* jumps from January to March 1799.

56

Kenneth Curry , ‘The monthly magazine’, in A. Sullivan (ed.), British Literary Magazines: Volume 2, the Romantic Age, 1789–1836 (Westport, Connecticut: Greenwood Press, 1983), 314–19.

57
*Monthly Magazine* 1 (1796), 144, 237, 322–5, 413.

58
*Monthly Magazine* 6 (1798), 140–1.

59
*Ibid.*, 51, 142, 190, 210, 238, 293, 372, 377, 473. Perkins’s Tractors were patented metal alloy strips passed over the skin to relieve pains and cutaneous conditions.

60
*Ibid.*, 25, 26, 241, 425.

61 ‘To the Public’, *Medical and Physical Journal*, 1 (1799), 312.

62
*Medical and Physical Journal*, 4 (1800), I, 572.

63 Cumbria Archives, Whitehaven, Day Books of John Ware, DA276A; Alan Mackintosh , The Patent Medicines Industry in Georgian England: Constructing the Market by the Potency of Print (London: Palgrave Macmillan, 2018), 33, 139 .

64
*A General State of the Whitehaven Dispensary for the Year 1801* (Whitehaven: John Ware, 1802), 26.

65
*Loan Register of the Liverpool Medical Library, 1800–26*, Liverpool Medical Institution, Liverpool; *Register of Circulation of the Leeds General Infirmary Library*, *1802–17*, Special Collections, University of Leeds, MS 2032/1.

66
*Medical and Physical Journal*, 4 (1800), 380.

67
*Ibid.*, 3 (1800), 270, 473; *op. cit.*, 4 (1800), 83.

68 ‘Advertisement’, *Medical and Physical Journal*, 3 (1800), i.

69 Chalmers, *op. cit.* (note 7), 39; ‘Preface’, *London Medical Journal*, 1 (1781), iii.

70 Zachs, *op. cit. (* note 24), 180.

71
*Ibid.*, 181.

72 Topham, *op. cit.* (note 9), 119–52: 141.

73
*Medical and Physical Journal*, 24 (1810), 265.

74 Curry, *op. cit.* (note 56).

75
*Medical and Physical Journal*, 1 (1799), 518.

76 ‘Advertisement’, *op.cit.* (note 68).

77
*Medical and Physical Journal*, 3 (1800), 128.

78
*Ibid.*, 192, 288, 588.

79
*Ibid.*, 3 (1800), 6, 221, 333, 421, 447, 459, 516, 529, 536; *op. cit.*, 4 (1800), 3, 156, 320, 441, 487.

80
*London Medical Review*, 3 (1800), i; *op. cit.*, 4 (1800), 404; *op. cit.*, 5 (1800), iii.

81
*Medical and Physical Journal*, 5 (1801), 591.

82
*London Medical Review*, 8 (1802), iii–iv.

83
*Register of Circulation*, *op.cit.* (note 65).

84 Cumbria Archives, *op.cit.* (note 63).

85
*Medical and Physical Journal*, 4 (1800), 21–4.

86

Irvine Loudon , Medical Care and the General Practitioner 1750–1850 (Oxford: Clarendon Press, 1986), 1–38, 94–9; Thomas Neville Bonner , Becoming a Physician: Medical Education in Britain, France, Germany and the United States, 1750–1945 (Oxford: Oxford University Press, 1995), 57 ; Robert Masters Kerrison , An Inquiry into the Present State of the Medical Profession in England (London: Longman, Hurst, Rees, Orme and Brown,1814), 31–2; Peter Puzzle-Pate , ‘Medical Science Exemplified’, The Scourge, 2 (1811), 260–8: 261; Susan Lawrence , Charitable Knowledge: Hospital Pupils and Practitioners in Eighteenth-Century London (Cambridge: Cambridge University Press, 1996); Christelle Rabier , ‘Medicalizing the surgical trade, 1650–1820: workers, knowledge, markets and politics’, in T. Schlick (ed.), The Palgrave Handbook of the History of Surgery (London: Palgrave Macmillan, 2018), 54–70 .

87

Michael Brown , Emotions and Surgery in Britain, 1793–1912 (Cambridge: Cambridge University Press, 2022), 19–21 ; Rabier, *op. cit.* (note 86), 57.

88
*Medical and Physical Journal*, 4 (1800), 219.

89

Michael Bennett , War against Smallpox: Edward Jenner and the Global Spread of Vaccination (Cambridge: Cambridge University Press, 2020), 79 .

90
*Medical and Physical Journal*, 4 (1800), i.

91 Bennett, *op. cit.* (note 89), 100–2; *Medical and Physical Journal*, 1 (1799), ii. Although Jenner received a St Andrews MD in 1792, he trained as a surgical pupil of John Hunter.

92 Topham, *op. cit.* (note 9); Dawson, *Scientific Communities*, *op. cit.* (note 9).

93 Dawson, *Scientific Periodicals*, *op. cit.* (note 9), 41.

94

Alex Csiszar , The Scientific Journal: Authorship and the Politics of Knowledge in the Nineteenth Century (London: University of Chicago Press, 2018), 6; Iain Watts , ‘“We Want No Authors”: William Nicholson and the Contested Role of the Scientific Journal in Britain, 1797–1813’, British Journal for the History of Science, 47 (2014), 397–419: 409; Dawson, *Scientific Periodicals*, *op.cit.* (note 9), 41.

95 Dawson, *Scientific Communities*, *op. cit.* (note 9); Csiszar, *op. cit.* (note 94), 6–8; Fyfe, *op. cit.* (note 16), 98.

96 Csiszar, *op. cit.* (note 94), 48; Fyfe, *op. cit.* (note 16), 89: Watts, *op. cit.* (note 94), 405.

97 Topham, *op. cit.* (note 9), 149; Watts, *op. cit.* (note 94), 405.

98 Three of the six were London physicians, and three held Edinburgh academic posts. None earned most of their income from their periodicals.

99
*Medical and Physical Journal*, 5 (1801), 108–110.

100 Dawson, *Scientific Communities*, *op. cit.* (note 9); Loudon, *op. cit.* (note 6); Carin Berkowitz , Charles Bell and the Anatomy of Reform (London: University of Chicago Press, 2015), 81 .

101 Watts, *op. cit.* (note 94), 406.

102 Brown, *op. cit.* (note 7); Berkowitz, *op. cit.* (note 100), 86.

103
*Medical and Physical Journal*, 8 (1802), 127; *op. cit.*, 10 (1803), 165, 258, 347.

104

Alan Mackintosh , ‘Warfare and the Launch of Medical Reform in Britain, 1793–1811’, Medical History, 65 (2021), 267–85.

105
*Medical and Physical Journal*, 15 (1806), 93.

106
*Ibid.*, 18 (1807), 1.

